# Pericentric inversion in chromosome 2 and their fertility issues: three cases report and literature review

**DOI:** 10.1515/med-2026-1408

**Published:** 2026-07-06

**Authors:** Wenjie Tian, Ranwei Li, Xiuyan Wang

**Affiliations:** Department of Urology, The Second Hospital of Jilin University, Changchun, Jilin, China

**Keywords:** breakpoint, genetic counseling, male infertility, pericentric inversion

## Abstract

**Objectives:**

Genetic counseling remains challenging for carriers of chromosomal inversions due to variable reproductive outcomes. This study aims to characterize the clinical features of three male carriers with chromosome 2 inversion.

**Methods:**

All participants underwent comprehensive assessment, including karyotype analysis and semen examination. Relevant cases were retrieved via PubMed, and candidate genes at chromosome 2 breakpoints were analyzed.

**Results:**

xCytogenetic analysis identified three karyotypes: 46,XY,inv(2)(p15q13), 46,XY,inv(2)(p13q11), and 46,XY,inv(2)(p11.2q13). A total of 22 previously reported cases were included for comparative analysis. inv(2)(p11q13) and inv(2)(p11.2q13) were the most prevalent karyotypes. Breakpoints at 2p11, 2p11.2, and 2q13 appeared to have minimal impact on spermatogenesis, while breakpoints at 2p13 and 2q11 may impair spermatogenesis by disrupting the structure and function of relevant genes located at 2p23, 2p13, 2q11, and 2q33.

**Conclusions:**

Detailed characterization of inversion breakpoints is essential for providing precise genetic counseling to carriers of chromosome 2 inversion.

## Introduction

One of the main contributing factors to male infertility, a disorder with several facets, is genetic etiology [1], 2]. Chromosome inversion is a major contributing factor to this pathogenesis [3], 4]. In clinical situations, inverted carriers may undergo preimplantation genetic diagnosis (PGD) to produce healthy babies [5], 6]. However, as several investigations have shown, the effectiveness of PGD in helping inverted carriers is still debatable [7]. The type of inversion has no effect on the embryo’s condition. Natural conception combined with a prenatal diagnosis may be possible for couples with inverted carriers who get genetic counseling [8]. The provision of genetic counseling is still significantly hampered by the diversity of reproductive results among inverted carriers.

Recent studies have investigated the association between chromosomal inversions and male infertility. The critical breakpoint of chromosome 1 inversion has been shown to affect spermatogenesis and is associated with azoospermia [9]. In addition, the 6p21 breakpoint in chromosome 6 inversion has been linked to asthenospermia [4]. The relationship between chromosome 10 inversion and male fertility has also been discussed [10]. Chromosome 9 pericentric inversion, once regarded as a benign polymorphism, may be associated with poor semen quality resulting from impaired spermatogenesis [11]. Accordingly, genetic counseling is recommended for inv(9) carriers during early pregnancy and in adulthood [12]. Further studies are warranted to clarify the impact of chromosome 2 inversion on male fertility.

In the present study, we found that the breakpoints 2p11, 2p11.2, and 2q13 may not substantially affect spermatogenesis. By contrast, breakpoints at 2p13 and 2q11 on chromosome 2 may disrupt the structure and function of relevant genes, consequently resulting in reduced sperm production. Nonetheless, carriers of these inversions display a wide spectrum of clinical outcomes.

## Materials and methods

### Study design and settings

This observational retrospective study was conducted at The Second Hospital of Jilin University.

### Patients

Three male patients who presented to the andrology clinic were enrolled in this study. A standardized questionnaire was used to collect detailed information, including medical history, fertility history, family genetic history, smoking and alcohol consumption, radiation exposure, infectious history, and other relevant factors. A thorough physical examination was performed for each patient, with height, weight, testicular volume and other parameters recorded. Given the obtained informed consent, basic hormonal measurements and molecular genetic analyses were not conducted. Meanwhile, the relevant clinical data of the patients and their spouses were also documented.

### Semen analysis

Semen analysis was performed according to the criteria recommended by the World Health Organization (WHO) [13]. Semen samples were collected by masturbation after 3–7 days of sexual abstinence. After complete liquefaction, sperm concentration, total motility, and progressive motility were evaluated using a computer-aided sperm analysis system (Shanghai Beion Medical Technology Co., Ltd., Shanghai, China). Sperm morphology was assessed by two qualified technicians following Diff-Quik staining. Notably, azoospermia was confirmed in all patients, as no spermatozoa were detected in at least two separate semen analyses.

### Cytogenetic analysis

Peripheral blood samples (2 mL) were collected from each patient into sterile heparin-containing tubes. Lymphocytes were cultured in 5 mL of commercial medium supplemented with 2 % phytohemagglutinin (Yishengjun; Guangzhou Baidi Biotech, Guangzhou, China) at 37 °C for 72 h. Prior to harvest, cultured lymphocytes were treated with 0.1 mL of colcemid for 30 min. Harvesting and G-banding staining were performed according to standard protocols. Karyotype results were described in accordance with the International System for Human Cytogenetic Nomenclature (ISCN 2020).

### Literature review

A literature search was conducted in PubMed for studies on chromosome 2 inversion published between 1980 and 2024, to explore the correlation between this chromosomal abnormality and male infertility. Only cases of male infertility were included, with those involving female infertility, hematological disorders, and fetal prenatal diagnosis excluded. Additionally, the Online Mendelian Inheritance in Man (OMIM; https://www.ncbi.nlm.nih.gov/omim) database was queried to identify chromosome 2-related genes, thereby investigating the association between inversion breakpoints and male infertility.

### Ethical statement

Approval for this investigation was granted by the Second Hospital of Jilin University’s Ethics Committee (2026-082). The requirement for informed consent was waived by the Ethics Committee due to the retrospective study design.

## Results

### Patient characteristics

**Figure 1:**
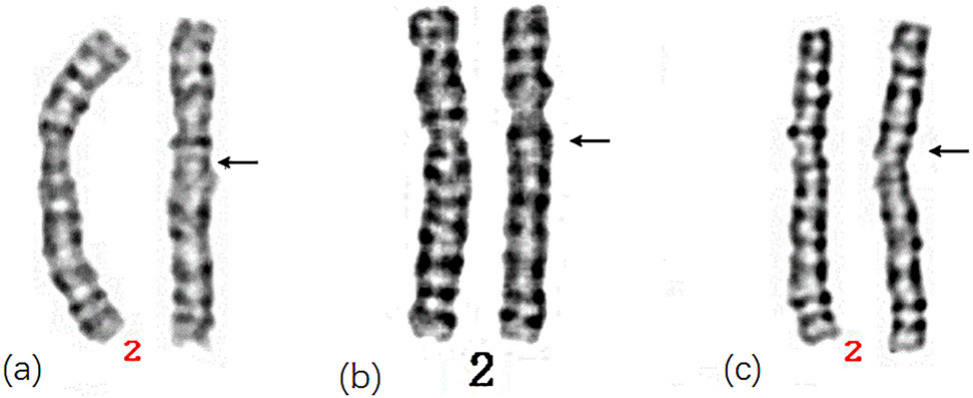
G-banding karyotypes of the three patients in this study.

Three male patients carrying chromosome 2 inversion were enrolled in this study. The clinical data of the patients and their spouses are summarized in Table 1. Patient 1 exhibited a normal phenotype and normal semen parameters. His karyotype was identified as 46,XY,inv(2)(p15q13) (Figure 1a), which was detected after his 3-year-old daughter was diagnosed with a chromosomal abnormality. Patient 2 presented with a normal phenotype and had a 6-year history of infertility. Semen analysis confirmed azoospermia, and cytogenetic analysis revealed a karyotype of 46,XY,inv(2)(p13q11) (Figure 1b). Following genetic counseling, the couple opted for artificial insemination with donor sperm, and a healthy child was subsequently born. Patient 3 had a normal phenotype and normal semen parameters. His karyotype was determined as 46,XY,inv(2)(p11.2q13) (Figure 1c), which was detected during prenatal fetal chromosome analysis of his wife. The fetus inherited the same inversion [46,XY,inv(2)(p11.2q13)] and showed normal development after birth. All three patients’ spouses had a normal karyotype (46,XX) with no abnormal findings on routine clinical examinations. After genetic counseling and obtaining informed consent, the parents of Patient 2 declined chromosomal analysis.

**Table 1:** Clinical profile of three patients.

Item	Case 1	Case 2	Case 3
Age, years	31	34	23
Karyotype	46,XY,inv(2)(p15q13)	46,XY,inv(2)(p13q11)	46,XY,inv(2)(p11.2q13)
Semen volume, mL	2.0	2.2	1.8
Sperm concentration, 10^6^ per mL	22	0	33
Total motility, %	45	0	42
Progressive motility, %	36	0	35
Sperm morphology, normal forms, %	8	0	10
Karyotype of spouse	46,XX	46,XX	46,XX
Routine genomic examination of spouse	No abnormal changes were observed	No abnormal changes were observed	No abnormal changes were observed

### Summary of reported literature data

A total of 22 cases with pericentric inversion of chromosome 2 were retrieved via literature review to assess the association between this chromosomal abnormality and male infertility. The reproductive history and semen parameters of these inversion carriers, including the three patients from the present study, are summarized in Table 2. Among these cases, 11 carried inv(2)(p11q13) and 10 carried inv(2)(p11.2q13). Although some carriers exhibited abnormal semen characteristics, many presented with infertility, recurrent spontaneous abortion, or normal reproductive outcomes in their offspring. Notably, Patient 2 in this study had azoospermia and a karyotype of 46,XY,inv(2)(p13q11).

**Table 2:** Clinical features of male carriers involving chromosome 2 inversion.

Case	Karyotype	Seminal parameters	Reproductive history of the couple	Reference
1	inv(2)(p11q13)	Severe oligoasthenozoospermia	Infertility	Morel et al. [14]
2	inv(2)(p11q13)	N/A	Recurrent abortion	Portnoï et al. [15]
3	inv(2)(p11q13)	N/A	Recurrent abortion	Portnoï et al. [15]
4	inv(2)(p11q13)	N/A	Recurrent abortion	Portnoï et al. [15]
5	inv(2)(p11q13)	N/A	ICSI	Testart et al. [16]
6	inv(2)(p11q13)	N/A	ICSI	Testart et al. [16]
7	inv(2)(p11q13)	N/A	Spontaneous abortion	Srebniak et al. [17]
8	inv(2)(p11q13)	Normozoospermia	Infertility	Ferfouri et al. [18]
9	inv(2)(p11q13)	Normozoospermia	Familial study after a prenatal diagnosis	Ferfouri et al. [18]
10	inv(2)(p11q13)	N/A	Having 2 children with normal phenotypes	Djalali et al. [19]
11	inv(2)(p11q13)	N/A	A son carrying inv (2)(p11q13)	Baccichetti et al. [20]
12	inv(2)(p11.2q13)	N/A	Repeated miscarriage	Goddijn et al. [21]
13	inv(2)(p11.2q13)	Oligoasthenospermia	N/A	Perrin et al. [22]
14	inv(2)(p11.2q13)	Severe oligozoospermia	NI	Dul et al. [23]
15	inv(2)(p11.2q13)	Oligoasthenozoospermia	IVF-PGS	Ramos et al. [24]
16	inv(2)(p11.2q13)	N/A	Have a phenotypically abnormal child	Lacbawan et al. [25]
17	inv(2)(p11.2q13)	Oligoasthenospermia	N/A	Anton et al. [26]
18	inv(2)(p11.2q13)	N/A	Familial carrier	Fickelscher et al. [27]
19	inv(2)(p11.2q13)	Teratospermia	Preimplantation genetic diagnosis	Escudero et al. [28]
20	inv(2)(p11.2q13)	N/A	N/A	Anton et al. [29]
21	inv(2)(p21q14.2)	Normal sperm concentration	NI	Dul et al. [23]
22	inv(2)(p23q33)	N/A	N/A	Mikhaail-Philips et al. [30]
23	inv(2)(p15q13)	Normal semen quality	A daughter carrying inv (2)(p15q13)	This study
24	inv(2)(p13q11)	Azoospermia	Primary infertility	This study
25	inv(2)(p11.2q13)	Normal semen quality	Being found after a prenatal diagnosis	This study

N/A, not applicable; IVF-PGS, *in vitro* fertilization with preimplantation genetic screening; NI, chromosomal abnormality without increased risk for miscarriage or a child with congenital anomalies; ICSI, intracytoplasmic sperm injection.

To investigate the correlation between chromosome 2 breakpoints and clinical phenotypes, chromosome 2-related genes are summarized in Table 3. A total of six genes associated with male infertility were identified at the chromosome 2 breakpoints 2p23, 2p13, 2q11 and 2q33.

**Table 3:** Important genes and its functions related to the breakpoints on chromosome 2 in this study.

Breakpoint	Gene	Full name of gene	Loci	Expression or function	Clinical findings
2p23	DRC1 (615288)	Dynein regulatory complex, subunit 1	2p23.3	DRC1 is required for the structural stability of flagella.	Spermatogenic failure
	PFN4 (620046)	Profilin family, member 4	2p23.3	PFN4 is required during spermiogenesis and is required for male fertility	N/A
2p13	M1AP (619098)	Meiosis 1-associated protein	2p13.1	Highly expressed in the cytoplasm of spermatogonia and primary spermatocytes	Spermatogenic failure
	GMCL1 (618627)	Germ cell-less 1, spermatogenesis-associated	2p13.3	Highly expressed in primary spermatocytes	Azoospermia or asthenozoospermia
2q11	TSGA10 (607166)	Testis-specific protein 10	2q11.2	TSGA10 has a critical role in spermatogenesis.	Spermatogenic failure
2q33	C2CD6 (619776)	C2 calcium-dependent domain-containing protein 6	2q33.1	C2CD6 is required for sperm motility and fertilization.	Globozoospermia

N/A, not applicable.

## Discussion

Male infertility is closely associated with chromosomal abnormalities [31]. Chromosomal inversion represents one of the most common structural rearrangements, characterized by a 180° rotation and reinsertion of a chromosomal segment between two breakpoints. Carriers of chromosomal inversions frequently present with adverse reproductive outcomes, including infertility, recurrent miscarriage, and abnormal pregnancies [32], 33]. Although extensive research has been performed, the precise mechanisms underlying these reproductive impairments remain unclear. Several potential mechanisms have been proposed. First, the interchromosomal effect (ICE) has long been debated in inversion carriers [34], [35], [36], [37]. Initially, ICE was hypothesized to occur when structural abnormalities on one chromosome affect the behavior of other chromosomes or their segments. However, Ogur et al. [38] suggested that ICE is negligible or absent, and Young et al. [39] further demonstrated no significant interchromosomal effects in individuals carrying chromosomal inversions. Second, genomic instability induced by chromosomal inversions may directly contribute to male infertility [40]. Elevated sperm DNA fragmentation rates have been observed in inversion carriers, providing a plausible mechanism for impaired fertility [41]. Third, the identification of critical chromosomal breakpoints in recent studies offers important implications for genetic counseling of inversion carriers [4], 9], 11], 42]. Nevertheless, the correlation between distinct inversion breakpoints and corresponding clinical phenotypes still warrants further in-depth investigation.

In the present study, three male carriers of chromosome 2 pericentric inversion were identified to explore the association between this chromosomal abnormality and adverse reproductive outcomes (Table 1). The carrier with inv(2)(p15q13) had a child with a normal phenotype, whereas the patient with inv(2)(p13q13) presented with azoospermia. To further clarify the relationship between chromosome 2 inversion and male infertility, a total of 22 previously reported cases were collected and analyzed (Table 2). Combining these with our three cases, 25 cases were included in the combined analysis, among which 11 carried inv(2)(p11q13) and 10 carried inv(2)(p11.2q13). These findings suggest that breakpoints at 2p11, 2p11.2, and 2q13 may exert minimal effects on spermatogenesis. Based on the available evidence, such inversions are associated with a wide spectrum of clinical manifestations, including infertility, recurrent spontaneous abortion, and normal reproductive outcomes. As indicated in Table 2, future studies with more detailed clinical information are warranted.

The breakpoints on inverted chromosomes and their corresponding critical genes have garnered increasing attention [4], 9]. To explore the association between chromosome 2 breakpoints and clinical phenotypes, genes located at breakpoints 2p23, 2p13, 2q11, and 2q33 are summarized in Table 3. A total of six genes associated with male infertility were identified. The *DRC1* gene at 2p23.3 has been linked to spermatogenic failure [43]. The *PFN4* gene, which is essential for male fertility, is also mapped to 2p23.3 [44]. The *M1AP* gene at 2p13.1 is associated with spermatogenic impairment [45]. The *GMCL1* gene at 2p13.3 is closely related to azoospermia or asthenozoospermia [46]. The *TSGA10* gene at 2q11.2 plays a vital role in sperm development [47]. The *C2CD6* gene at 2q33.1 is strongly associated with globozoospermia [48]. Based on the present case observations and previously published data, we hypothesize that disruption of the *M1AP*, *GMCL1*, and/or *TSGA10* genes may contribute to the pathogenesis of azoospermia in case 2 of this study. Further sophisticated mechanisms may also be involved. Additional cases and further in-depth studies are warranted to validate these findings.

Collectively, our findings indicate that pericentric inversion of chromosome 2 may represent a contributing factor to male infertility. Chromosomal analysis is therefore recommended for individuals with abnormal semen parameters or those undergoing assisted reproductive technology.

This study is limited by the small number of included cases with characterized chromosome 2 breakpoints and the incomplete semen parameter data in some published reports. Furthermore, functional molecular genetic studies were not performed in the present investigation.

This retrospective study demonstrated that the two most prevalent karyotypes among carriers were inv(2)(p11q13) and inv(2)(p11.2q13). Breakpoints at 2p11, 2p11.2, and 2q13 may not substantially affect spermatogenesis. In contrast, breakpoints at 2p13 and 2q11 may disrupt the structure and function of relevant genes, consequently leading to decreased spermatogenesis. Nevertheless, individuals with these inversions exhibit a broad spectrum of clinical phenotypes. Thus, identification of the specific breakpoints involved in chromosome 2 inversion is of critical importance for genetic counseling.

## References

[1] Batiha O, Burghel GJ, Alkofahi A, Alsharu E, Smith H, Alobaidi B (2022). Screening by single-molecule molecular inversion probes targeted sequencing panel of candidate genes of infertility in azoospermic infertile Jordanian males. Hum Fertil.

[2] Akalin H, Sahin IO, Paskal SA, Tan B, Yalcinkaya E, Demir M (2024). Evaluation of chromosomal abnormalities in the postnatal cohort: a single-center study on 14,242 patients. J Clin Lab Anal.

[3] Andó S, Koczok K, Bessenyei B, Balogh I, Ujfalusi A (2022). Cytogenetic investigation of infertile patients in Hungary: a 10-year retrospective study. Genes.

[4] Fan H, Liu Z, Zhan P, Jia G (2022). Pericentric inversion of chromosome 6 and male fertility problems. Open Med.

[5] Shetty S, Nair J, Johnson J, Shetty N, J AK, Thondehalmath N (2022). Preimplantation genetic testing for couples with balanced chromosomal rearrangements. J Reprod Infertil.

[6] Xie P, Hu L, Tan Y, Gong F, Zhang S, Xiong B (2019). Retrospective analysis of meiotic segregation pattern and interchromosomal effects in blastocysts from inversion preimplantation genetic testing cycles. Fertil Steril.

[7] Shao Y, Li J, Lu J, Li H, Zhu Y, Jiang W (2020). Clinical outcomes of preimplantation genetic testing (PGT) application in couples with chromosomal inversion, a study in the Chinese Han population. Reprod Biol Endocrinol.

[8] Tong J, Jiang J, Niu Y, Zhang T (2022). Do chromosomal inversion carriers really need preimplantation genetic testing?. J Assist Reprod Genet.

[9] Li R, Fan H, Zhang Q, Yang X, Zhan P, Feng S (2020). Pericentric inversion in chromosome 1 and male infertility. Open Med.

[10] Zhang X, Shi Q, Liu Y, Jiang Y, Yang X, Liu R (2021). Fertility problems in males carrying an inversion of chromosome 10. Open Med.

[11] Mottola F, Santonastaso M, Ronga V, Finelli R, Rocco L (2023). Polymorphic rearrangements of human chromosome 9 and male infertility: new evidence and impact on spermatogenesis. Biomolecules.

[12] Xie X, Li F, Tan W, Tang J (2020). Analysis of the clinical features of pericentric inversion of chromosome 9. J Int Med Res.

[13] World Health Organization (2021). WHO laboratory manual for the examination and processing of human semen.

[14] Morel F, Laudier B, Guérif F, Couet ML, Royère D, Roux C (2007). Meiotic segregation analysis in spermatozoa of pericentric inversion carriers using fluorescence in-situ hybridization. Hum Reprod.

[15] Portnoï MF, Joye N, van den Akker J, Morlier G, Taillemite JL (1988). Karyotypes of 1142 couples with recurrent abortion. Obstet Gynecol.

[16] Testart J, Gautier E, Brami C, Rolet F, Sedbon E, Thebault A (1996). Intracytoplasmic sperm injection in infertile patients with structural chromosome abnormalities. Hum Reprod.

[17] Srebniak M, Wawrzkiewicz A, Wiczkowski A, Kaźmierczak W, Olejek A (2004). Subfertile couple with inv(2), inv(9) and 16qh+. J Appl Genet.

[18] Ferfouri F, Clement P, Gomes DM, Minz M, Amar E, Selva J (2009). Is classic pericentric inversion of chromosome 2 inv(2)(p11q13) associated with an increased risk of unbalanced chromosomes?. Fertil Steril.

[19] Djalali M, Steinbach P, Bullerdiek J, Holmes-Siedle M, Verschraegen-Spae MR, Smith A (1986). The significance of pericentric inversions of chromosome 2. Hum Genet.

[20] Baccichetti C, Lenzini E, Peserico A, Tenconi R (1980). Study on segregation and risk for abnormal offspring in carriers of pericentric inversion of the (p11 leads to q13) segment of chromosome 2. Clin Genet.

[21] Goddijn M, Joosten JH, Knegt AC, van derVeen F, Franssen MT, Bonsel GJ (2004). Clinical relevance of diagnosing structural chromosome abnormalities in couples with repeated miscarriage. Hum Reprod.

[22] Perrin A, Caer E, Oliver-Bonet M, Navarro J, Benet J, Amice V (2009). DNA fragmentation and meiotic segregation in sperm of carriers of a chromosomal structural abnormality. Fertil Steril.

[23] Dul EC, van Echten-Arends J, Groen H, Dijkhuizen T, Land JA, van Ravenswaaij-Arts CM (2012). Chromosomal abnormalities in azoospermic and non-azoospermic infertile men: numbers needed to be screened to prevent adverse pregnancy outcomes. Hum Reprod.

[24] Ramos L, Daina G, Del Rey J, Ribas-Maynou J, Fernández-Encinas A, Martinez-Passarell O (2015). Comprehensive preimplantation genetic screening and sperm deoxyribonucleic acid fragmentation from three males carrying balanced chromosome rearrangements. Fertil Steril.

[25] Lacbawan FL, White BJ, Anguiano A, Rigdon DT, Ball KD, Bromage GB (1999). Rare interstitial deletion (2)(p11.2p13) in a child with pericentric inversion (2)(p11.2q13) of paternal origin. Am J Med Genet.

[26] Anton E, Vidal F, Egozcue J, Blanco J (2006). Genetic reproductive risk in inversion carriers. Fertil Steril.

[27] Fickelscher I, Liehr T, Watts K, Bryant V, Barber JC, Heidemann S (2007). The variant inv(2)(p11.2q13) is a genuinely recurrent rearrangement but displays some breakpoint heterogeneity. Am J Hum Genet.

[28] Escudero T, Lee M, Stevens J, Sandalinas M, Munné S (2001). Preimplantation genetic diagnosis of pericentric inversions. Prenat Diagn.

[29] Anton E, Blanco J, Egozcue J, Vidal F (2005). Sperm studies in heterozygote inversion carriers: a review. Cytogenet Genome Res.

[30] Mikhaail-Philips MM, Ko E, Chernos J, Greene C, Rademaker A, Martin RH (2004). Analysis of chromosome segregation in sperm from a chromosome 2 inversion heterozygote and assessment of an interchromosomal effect. Am J Med Genet A.

[31] Fu M, Chen M, Guo N, Lin M, Li Y, Huang H (2023). Molecular genetic analysis of 1,980 cases of male infertility. Exp Ther Med.

[32] Balasar Ö, Zamani AG, Balasar M, Acar H (2017). Male infertility associated with de novo pericentric inversion of chromosome 1. Turk J Urol.

[33] Ghorbel M, Baklouti-Gargouri S, ElGhazel H, Zribi N, Ben Abdallah F, Cherif M (2013). Pericentric inversion of chromosome 12 [inv (12) (p12q12)] associated with idiopathic azoospermia in one infertile Tunisian man. Biochem Biophys Res Commun.

[34] Batanian J, Hulten MA (1987). Electron microscopic investigations of synaptonemal complexes in an infertile human male carrier of a pericentric inversion inv(1)(p32q42). Regular loop formation but defective synapsis including a possible interchromosomal effect. Hum Genet.

[35] Hajlaoui A, Slimani W, Kammoun M, Sallem A, Braham S, Bibi M (2018). Sperm fluorescent in situ hybridisation study of interchromosomal effect in six tunisian carriers of reciprocal and robertsonian translocations. Andrologia.

[36] Schinzel AA, Adelsberger PA, Binkert F, Basaran S, Antonarakis SE (1992). No evidence for a paternal interchromosomal effect from analysis of the origin of nondisjunction in down syndrome patients with concomitant familial chromosome rearrangements. Am J Hum Genet.

[37] Balasar Ö, Acar H (2020). Investigation of the interchromosomal effects in male carriers with structural chromosomal abnormalities using FISH. Turk J Urol.

[38] Ogur C, Kahraman S, Griffin DK, Cinar Yapan C, Tufekci MA, Cetinkaya M (2023). PGT for structural chromosomal rearrangements in 300 couples reveals specific risk factors but an interchromosomal effect is unlikely. Reprod Biomed Online.

[39] Young D, Klepacka D, McGarvey M, Schoolcraft WB, Katz-Jaffe MG (2019). Infertility patients with chromosome inversions are not susceptible to an inter-chromosomal effect. J Assist Reprod Genet.

[40] Aston KI, Carrell DT (2012). Emerging evidence for the role of genomic instability in male factor infertility. Syst Biol Reprod Med.

[41] Perrin A, Nguyen MH, Bujan L, Vialard F, Amice V, Guéganic N (2013). DNA fragmentation is higher in spermatozoa with chromosomally unbalanced content in men with a structural chromosomal rearrangement. Andrology.

[42] Liehr T, Kosayakova N, Schröder J, Ziegler M, Kreskowski K, Pohle B (2011). Evidence for correlation of fragile sites and chromosomal breakpoints in carriers of constitutional balanced chromosomal rearrangements. Balkan J Med Genet.

[43] Zhang J, He X, Wu H, Zhang X, Yang S, Liu C (2021). Loss of DRC1 function leads to multiple morphological abnormalities of the sperm flagella and male infertility in human and mouse. Hum Mol Genet.

[44] Umer N, Phadke S, Shakeri F, Arévalo L, Lohanadan K, Kirfel G (2022). PFN4 is required for manchette development and acrosome biogenesis during mouse spermiogenesis. Development.

[45] Wyrwoll MJ, Temel ŞG, Nagirnaja L, Oud MS, Lopes AM, van der Heijden GW (2020). Bi-allelic mutations in M1AP are a frequent cause of meiotic arrest and severely impaired spermatogenesis leading to Male infertility. Am J Hum Genet.

[46] Kleiman SE, Yogev L, Gal-Yam EN, Hauser R, Gamzu R, Botchan A (2003). Reduced human germ cell-less (HGCL) expression in azoospermic men with severe germinal cell impairment. J Androl.

[47] Sha YW, Sha YK, Ji ZY, Mei LB, Ding L, Zhang Q (2018). TSGA10 is a novel candidate gene associated with acephalic spermatozoa. Clin Genet.

[48] Oud MS, Okutman Ö, Hendricks LAJ, de Vries PF, Houston BJ, Vissers LELM (2020). Exome sequencing reveals novel causes as well as new candidate genes for human globozoospermia. Hum Reprod.

